# Intensified Tuberculosis Case Finding among Malnourished Children in Nutritional Rehabilitation Centres of Karnataka, India: Missed Opportunities

**DOI:** 10.1371/journal.pone.0084255

**Published:** 2013-12-16

**Authors:** Prashant G. Bhat, Ajay M. V. Kumar, Balaji Naik, Srinath Satyanarayana, Deepak KG, Sreenivas A. Nair, Suryakanth MD, Einar Heldal, Donald A. Enarson, Anthony J. Reid

**Affiliations:** 1 Tuberculosis Control Unit, World Health Organisation, Country Office for India, New Delhi, India; 2 Operational Research Unit, International Union Against Tuberculosis and Lung Diseases, South-East Asia Regional Office, New Delhi, India; 3 Tuberculosis Control Unit, State Tuberculosis Office, Bangalore, Karnataka, India; 4 Operational Research Unit, International Union Against Tuberculosis and Lung Diseases, Paris, France; 5 Operational Research Unit (LUXOR), Medecins sans Frontieres, Brussels-Luxembourg.; National Taiwan University, Taiwan

## Abstract

**Background:**

Severe acute malnutrition (SAM) is the most serious form of malnutrition affecting children under-five and is associated with many infectious diseases including Tuberculosis (TB). In India, nutritional rehabilitation centres (NRCs) have been recently established for the management of SAM including TB. The National TB Programme (NTP) in India has introduced a revised algorithm for diagnosing paediatric TB. We aimed to examine whether NRCs adhered to these guidelines in diagnosing TB among SAM children.

**Methods:**

A cross-sectional study involving review of records of all SAM children identified by health workers during 2012 in six tehsils (sub-districts) with NRCs (population: 1.8 million) of Karnataka, India.

**Results:**

Of 1927 identified SAM children, 1632 (85%) reached NRCs. Of them, 1173 (72%) were evaluated for TB and 19(2%) were diagnosed as TB. Of 1173, diagnostic algorithm was followed in 460 (37%). Among remaining 763 not evaluated as per algorithm, tuberculin skin test alone was conducted in 307 (41%), chest radiography alone in 99 (13%) and no investigations in 337 (45%). The yield of TB was higher among children evaluated as per algorithm (4%) as compared to those who were not (0.3%) (OR: 15.3 [95%CI: 3.5-66.3]). Several operational challenges including non-availability of a full-time paediatrician, non-functioning X-ray machine due to frequent power cuts, use of tuberculin with suboptimal strength and difficulties in adhering to a complex diagnostic algorithm were observed.

**Conclusion:**

This study showed that TB screening in NRCs was sub-optimal in Karnataka. Some children did not reach the NRC, while many of those who did were either not or sub-optimally evaluated for TB. This study pointed to a number of operational issues that need to be addressed if this collaborative strategy is to identify more TB cases amongst malnourished children in India.

## Introduction

Severe acute malnutrition (SAM) is a major public health problem, especially among under-five children in developing countries [1]. Owing to reduced immunity [2], children with SAM are at a higher risk of acquiring infectious diseases including Tuberculosis (TB) [3-5], which is a major contributor to high mortality among these children [6,7]. 

India accounts for one third of the global burden of malnourished children [8] and approximately 46% of the under-five population in the country are moderate to severely underweight (thin for age), 38% are moderately to severely stunted (short for age) and 19% moderately to severely wasted (thin for height) [9,10]. TB is one of the most important infectious diseases in India, with almost 40% of the population infected, approximately three million prevalent active tuberculosis cases (256/100,000 population) and two million incident cases every year (185/100,000 population) [11]. It is well known that malnutrition is one of the strong risk factors for progression from TB infection to disease and contributes to about 26% of incident TB globally [12-15]. Hence, the World Health Organization (WHO) recommends intensified case finding for TB amongst malnourished children [16]. 

Recently, the Ministry of Health, Government of India, established several Nutritional Rehabilitation Centres (NRCs) across the country to comprehensively manage SAM children under its National Rural Health Mission initiative. SAM children identified at the villages are being routinely referred to these NRCs for comprehensive management. These specialised clinics are staffed by a paediatrician and equipped to effectively manage SAM children and diagnose infectious diseases, including TB. 

Karnataka, a State in south India with a population of 60 million, has 16 such NRCs. Approximately 30% of the under-five population in the State are malnourished [10,17,18], 43% are stunted (short for age) and 17% wasted (thin for height) [10] and the mortality in this sub-group is quite high. In 2011, there was a public outcry concerning the appropriateness of the management of malnourished children by the State’s public health system. Eventually, a petition was filed in the State’s High Court requesting judicial examination of the process of evaluation and management of the malnourished children. Consequently, in 2012, the Court directed the health system to medically re-evaluate all SAM children for co-morbidities and provide appropriate medical care (including TB) to reduce morbidity and mortality in this sub-group of children [19,20]. 

In 2012, the National Tuberculosis Programme (NTP) in India, issued a new paediatric TB management guideline. This guideline included a new algorithm for suspecting and diagnosing TB in children [21]. As per this guideline, all malnourished children were eligible for TB evaluation. In the context of the Karnataka court decision, this meant that all SAM children had to undergo re-evaluation for TB as per the new paediatric guideline.

Previously, there has been no evaluation of the application of this diagnostic guideline in the context of malnutrition. In addition, we could not find any published studies providing information on systematic screening for TB in malnourished children, in a programmatic setting. The circumstances in Karnataka provided an opportunity to assess both these aspects.

Hence we undertook an operational research study to evaluate the process and outcomes of TB screening among SAM children attending NRCs in Karnataka. The specific objectives were to ascertain the 1) proportion of identified SAM children who reached NRCs and were evaluated for TB as per the new paediatric TB diagnostic algorithm, 2) proportion found to have active TB disease and initiated on TB treatment and 3) association of demographic characteristics with these findings.

## Methods

### Ethics

Ethics approval was obtained from the Ethics Advisory Group (EAG) of the International Union Against Tuberculosis and Lung Disease (The Union), Paris, France. Since the study was a record review of the routinely collected programme data, without direct involvement of any children, written consent from the parents/guardians of SAM children evaluated was not considered necessary. The same was reviewed by the ethics committee (EAG of The Union) and the need for individual informed consent from the study participants was waived. In addition, written permission was obtained from the State TB programme authorities (Karnataka) for conducting the study.

### Design

Descriptive study involving retrospective review of routinely-collected programme data and medical records.

### Setting

We conducted the study in six Tehsils (sub-districts) of Karnataka State, India, with established NRCs, covering a total population of approximately 1.8 million. These NRCs were purposively selected to represent varied combinations of workload, infrastructure and availability of human resources. The selected NRCs included one from district headquarters having an inpatient facility and high workload. Two others did not have a full time paediatrician, but a temporary arrangement was made to have a paediatrician from a nearby public hospital visit these centres on fixed days of the week. On average, full strength electricity (required for chest radiography) was available for only three hours during the day (working hours) among all six NRCs but two had a generator to provide backup power when needed. Public transport to the NRCs varied widely, from very good in two to moderate to poor in the rest. 

SAM children are defined as those with a low weight to height ratio (less than three standard deviations) identified at the villages by the health workers using a ‘WHO weight for height growth chart for 6-59 months’ at each visit [22]. Identified SAM children were referred to the NRCs that were equipped for TB diagnosis, specifically with tuberculin skin testing, using PPD (purified protein derivative of RT23 or equivalent) and chest radiography facilities. Paediatricians at these centers were trained on the new NTP guideline for the management of TB among children. These NRCs had facilities for conducting sputum smear microscopy. The new diagnostic guidelines in India [21] required that presumptive paediatric TB patients undergo either a sputum examination or the combination of PPD testing and chest radiograph, if a sputum specimen could not be obtained [21]. The guideline specified 2TU as optimal PPD strength for tuberculin skin testing, to be read after 48-72 hours, with a cut off for significant induration of five mm in SAM children.

Each peripheral health worker in Karnataka maintained a register of all SAM children identified in their area during the calendar year. SAM children thus identified were referred to NRCs with a referral slip mentioning the name of the sub-center (health care facility at villages staffed by a health care worker) and a unique identifier for each referred child. When these children reached the NRCs, this information was entered into the registers at the NRCs. NRC registers also captured data on TB evaluation status, the methods used and the outcomes of evaluation including initiation of treatment.

### Sampling and study period

We collected information on all SAM children listed in the registers of all villages in the study tehsils from January-December 2012. The data were collected during the period November 2012 to January 2013. 

### Data sources, variables and data collection procedure

We used the registers maintained by the peripheral health workers to obtain a list of all children identified and referred. We used the NRC registers to assess what proportion reached the NRCs and underwent TB screening procedures. We defined a child as having followed the proscribed diagnostic algorithm if either his/her sputum was examined for TB and/or if he/she was evaluated with the combined tests of PPD and radiography. 

### Data management and analysis

The data were systematically collected on a standardized data collection form and 10% were re-verified for transcription errors. The data were doubly entered electronically into a predesigned and tested EpiData file (Version 3.1, EpiData Association www.epidata.dk), validated for consistency and the data finalized. The data were analyzed using EpiData software [Version V2.2.2.182] Frequencies and proportions were calculated for each variable. Differences between groups were compared using Chi square tests and their 95% confidence intervals. A P value of <0.05 was considered statistically significant. 

## Results

**Figure 1:**
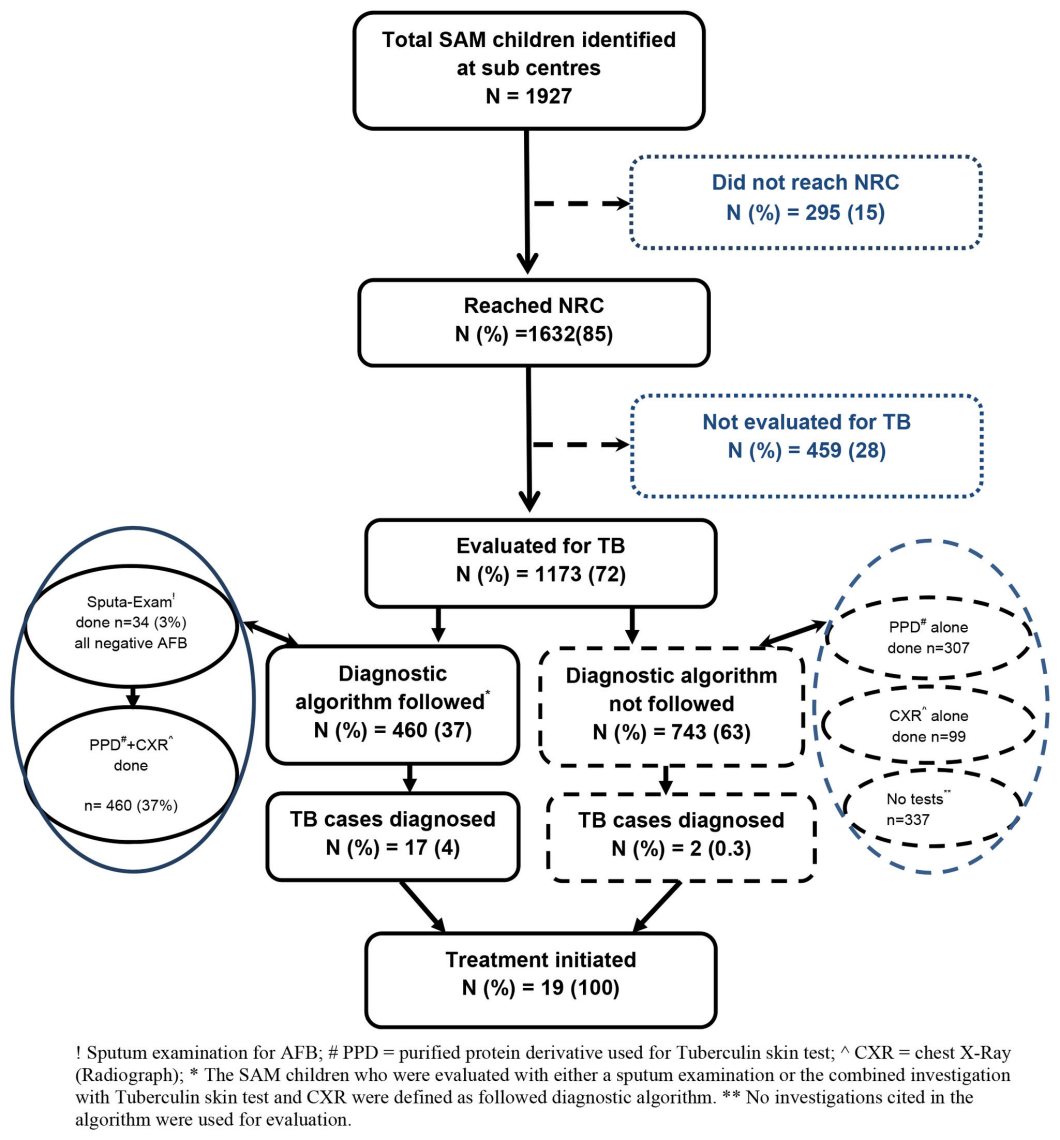
Tuberculosis screening for severe acute malnourished children. Intensified tuberculosis case finding among Severe Acute Malnourished (SAM) children at Nutritional Rehabilitation Centres (NRCs) of six selected Tehsils (sub-districts) of Karnataka, 2012.

In 2012, 1927 SAM children were identified at villages of the six study tehsils. Of these, the proportion who reached NRC, the proportion evaluated for and diagnosed with TB and initiated on treatment is shown in Figure **1**. Overall, 15% of the identified SAM children did not reach the NRC to which they were referred. Of those who reached the NRC, 28% were not evaluated for TB and among those who were evaluated, 63% children were not evaluated according to the NTP diagnostic guidelines. In children who were evaluated as per the new NTP diagnostic algorithm, 4% were diagnosed with TB when compared to 0.3% in children who were not evaluated as per the diagnostic algorithm. This difference was statistically significant (OR: 15.3 [95%CI: 3.5-66.3]). Overall, 2% SAM children evaluated were diagnosed for TB.

**Figure 2:**
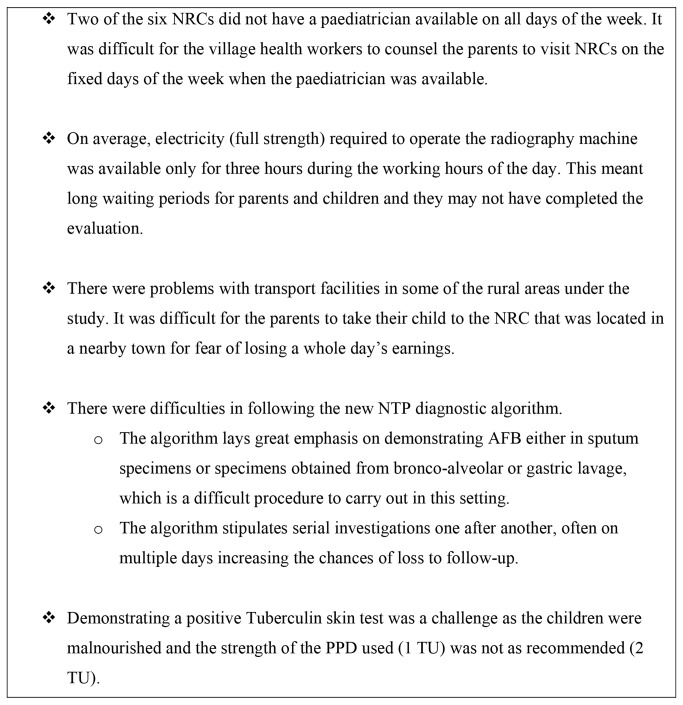
Challenges observed in application of NTP diagnostic algorithm in NRCs.

The outcome of TB screening in relation to various demographic characteristics is shown in Table **1**. Of the identified SAM children two thirds were female. With respect to age, a smaller proportion of children aged <11 months was evaluated for TB as per the diagnostic algorithm, when compared to the reference population of 12-35 months (OR: 0.53 [95%CI: 0.34-0.83]). In terms of residential status, the proportion of urban children reaching NRCs was higher than rural children (OR: 2.42 [95%CI: 1.8-3.1]). However, the proportion evaluated for TB was higher in rural children (OR: 6.57 [95% CI: 5.1-8.5]). With respect to NRC sites, the proportion of children who reached the NRCs was relatively lower at one site (K01- 43%) and a relatively lower proportion of children were evaluated for TB at two other sites (G01 and R01). Evaluation as per NTP guidelines was carried out well at only one site, (C01-95%) which alone contributed to >75% of the total TB cases detected. The key challenges in adhering to the NTP diagnostic algorithm that were identified by the study investigators during the data collection process are given in Figure **2**.

**Table 1:** Results of TB screening among Severe Acute Malnourished (SAM) children in relation to demographic characteristics in six Tehsils^*$*^ of Karnataka, India, January-December 2012.

**Variables**	**Number of children identified at the villages**	**Number (%) reached NRC**	**Number (%) evaluated for TB among ‘B’**	**Number (%) followed diagnostic algorithm among ‘C’**	**Number (%) with TB among ‘C’**
	**A**	**B**	**C**	**D**	**E**
**Total**	**1927**	**1632 (85)**	**1173 (72)**	**430 (37)**	**19 (2)**
**Sex**					
Male	768	654 (85)	477 (73)	176 (37)	5 (1)
Female	1159	978 (84)	696 (71)	254 (37)	14 (2)
**Age (months)**					
0-11	189	165 (87)	128 (78)	25 (19)**^*^*^**	0 (0)
12-35	1010	857 (85)	577 (67)	226 (39)	10 (2)
36-60	727	609 (84)	468 (74)	179 (38)	9 (2)
**Residence**					
Urban	869	787 (91)	425 (54)	156 (37)	4 (1)
Rural	1058	845 (80)	748 (88)^*^	274 (37)	15 (2)
**Study site**					
G01	872	821 (93)	397 (48)	130 (33)	0 (0)
R01	63	44 (70)	26 (59)	12 (46)	1 (4)
C01	341	268 (79)	268 (100)	255 (95)	15 (6)
K01	266	114 (43)	114 (100)	0 (0)	0 (0)
H01	317	317 (100)	300 (95)	0 (0)	0 (0)
U01	68	68 (100)	68 (100)	33 (48)	3 (4)

SAM=Severe Acute Malnourished; NRC=Nutritional Rehabilitation Centre; TB=Tuberculosis; OR=Odds Ratio; CI=Confidence Interval; $Tehsil=sub-district block in Karnataka; #SAM children evaluated for TB with sputum microscopy and/or Tuberculin skin test+Radiography are considered as followed diagnostic algorithm; ;*OR 6.57 (95% CI: 5.1-8.5); ^ OR 1.9 (95% CI: 1.2-3)

## Discussion

To our knowledge, this is the first study in India reviewing the application of the new NTP diagnostic algorithm for diagnosing TB in malnourished children. Overall, few children benefited from the introduction of the new diagnostic criteria and relatively few new cases of TB were identified – indicating a large “missed opportunity” in preventing morbidity and mortality among these children. 

This study has certain strengths. All children initially identified as SAM were included and all were accounted for at the end of the study. The NRCs involved were chosen purposively to cover a wide range of circumstances, in terms of human resources, equipment and environment. Having recent, up to date evaluations for all the children, thanks to re-evaluation of children as per the Court order, meant a more consistent set of clinical data. 

Several limitations of this study are also acknowledged. First, as with all record review studies, this study is limited by the quality of the data that was recorded. Precautions were taken with enhanced data supervision, which involved cross checking from multiple sources to mitigate the possible errors of recording. Second, the study was designed to obtain quantitative information in short span of time and some of the issues and challenges that were observed during the study, because of its importance and relevance have been reported as such without an in-depth assessment. Third, none of the diagnosed TB patients were bacteriologically confirmed and none were processed for culture. 

The proportions of children reaching NRCs varied across sites. While definite reasons for this were unknown, the study finding that a relatively lower proportion of children from rural areas reached NRCs (that are located in urban areas) when compared to urban areas indicated that NRCs were not easily accessible to the rural populations. Another possible reason could be that, since severely malnourished children are at high risk of mortality, there could have been deaths, which we were not able to pick up through this record review study. Interestingly, one site contributed to >75% of the total cases detected. While the exact reason for the same is not clear, availability of the full time paediatrician and availability of functional radiography could have contributed to better performance. 

It was unfortunate that nearly a third of the children who reached NRCs were not evaluated for TB and among those evaluated, the NTP diagnostic algorithm was not followed in two thirds. There were issues with logistics support and difficulties in evaluating the children as per the diagnostic algorithm stipulated by the NTP. This resulted in only 2% of the children who were evaluated being diagnosed as TB. This was low in comparison to the findings in other studies, where the proportion of TB diagnosed in severe malnourished children was 4-20% [7,23,24]. While we are not sure of the exact reasons, possible explanations for the low proportion of TB cases detected could be 1) inappropriate strength of the PPD used for the diagnosis of TB. NTP guidelines stipulate that a PPD strength of 2TU be used. However, PPD of strength 1TU was supplied by the Ministry of Health and 2TU was not available, 2) non-adherence to the cut off values for induration of the PPD test. As per the guidelines, induration of 5mm indicates TB infection in SAM cases [21] and this might not had been used correctly in some of our study setting as per the information given by supervisory staff, 3) difficulties in obtaining chest radiography among children specifically because of irregular power supply resulting in many children not having undergone chest radiography. 4) it was possible that there had not been enough time for training the staff and paediatricians to be comfortable with the new protocol before its introduction, 5) some clinics might have been too busy for proper evaluation, or the paediatrician was not always present, especially for those NRCs that had a visiting paediatrician on fixed days of the week. 

Given these gaps in implementation, some immediate steps should be undertaken: 1) provide support for travel to ensure that parents can take their malnourished children to the NRCs. 2) intensify advocacy, communication and social mobilisation activities in rural areas to optimise attendance at the NRCs 3) provide power generators with a capacity to run an X-Ray machine in sites with inadequate electricity 4) supply PPD of optimal strength (2TU) 5) supportive supervision and regular audit should be introduced to track progress and 6) possible further simplification of the diagnostic algorithm that would make screening and adhering to the guidelines easier. 

## Conclusion

This study showed that TB screening in severely malnourished children at NRCs was sub-optimal in Karnataka State in India. Some children did not reach the NRC, while many of those who did were either not screened or sub-optimally exposed to the screening algorithm. This study pointed to a number of operational issues that need to be addressed to identify more TB cases amongst malnourished children in India. 
